# Supplementary material for: Improving cryo-EM grids for amyloid fibrils using interface-active solutions and spectator proteins
Source: Biophys J. 2024 Feb 17;123(6):718–29. doi: 10.1016/j.bpj.2024.02.009 (PMC10995402; doi:10.1016/j.bpj.2024.02.009)
Supplement: Document S1. Figures S1–S5 and Tables S1 and S2 [file mmc1.pdf]

Biophysical Journal, Volume *123*

## Supplemental information

# Improving cryo-EM grids for amyloid fibrils using interface-active solutions and spectator proteins

**Dylan Valli, Saik Ann Ooi, Giorgio Scattolini, Himanshu Chaudhary, Alesia A. Tietze, and Michał Maj**

## SUPPLEMENTARY MATERIAL

### Cryo-EM data processing

All movies were motion-corrected and dose-weighted using RELION(1) own implementation of UCSF MotionCor2(2). The contrast transfer function (CTF) was estimated with CTFFIND4.1(3). The 3dem implementation of Topaz(4) for fibrils was used to train an auto-picking model based on 100 manually picked micrographs and all fibrils' start-end coordinates were automatically picked over the whole dataset. The particles were extracted with a box size of 960 pixels down-sampled to 240 px and an inter-box distance of 3 asymmetrical units of 4.75 Å. Several rounds of 2D classifications were carried out to separate the polymorphs. The classes with the dominant polymorph were selected and the particles were re-extracted with a 600 px box down-sampled to 300 px for further 2D classifications. The best particles were extracted at 320 box size and further separated in a 3D classification job without symmetry using a cylinder generated with relion_helix_toolbox as an initial model. The main resulting class showed a typical C2 symmetry and the corresponding particles were used to perform another round of 3D classification job with imposed C2 symmetry. The final particles were run through a 3D-refine job using the previous 3D class as an initial model and a narrow search for helical symmetry. Both half-maps converged to similar values for twist and rise of 178 degrees and 2.45 Å respectively. Finally, the particles were polished and a post-process job was run to obtain the final map with a resolution of 4.01 Å. The resolution was calculated using gold-standard FSC at 0.143 threshold.

### Model building

The published PDB model (PDB:7M64) was used as an initial model and fitted into the cryo-EM map in COOT(5). The model was refined in COOT and real space refinement in PHENIX(6) iteratively. The model and map were visualized with UCSF ChimeraX(7). The cryo-EM data collection, refinement, and validation statistics are summarized in Table S2.

Table S1: Surface tension values for milli-Q water, 100 mM HEPES, 20 mM Tris-HCl and 2 mM SDS

| | $\gamma$ / $mNm^{-1}$ |
|---|---|
| milli-Q water | 72.3±0.3 |
| HEPES 100 mM | 71.4±0.3 |
| Tris-HCl 20 mM | 71.7±0.5 |
| SDS 2 mM | 62.6±1.1 |

Table S2: Data collection and refinement parameters for hIAPP-WT double S polymorph.

| | WT P1 (EMDB-18887) (PDB 8R4I) |
|---|---|
| **Data collection and processing** | |
| Magnification | 105 000 |
| Voltage (kV) | 300 |
| Electron exposure ($e^-$/Å$^2$) | 39.926 |
| Defocus range (µm) | 0.5 to 1.7 |
| Pixel size (Å) | 0.828 |
| Symmetry imposed | C2 |
| Twist (°) | 178.25 |
| Rise (Å) | 2.46 |
| Initial particles images (no.) | 189867 |
| Final particles images (no.) | 10566 |
| Map resolution (Å) | 4.01 |
| **Refinement** | |
| Initial model used (PDB code) | 7M64 |
| Model resolution (Å) | 4.01 |
| FSC threshold | 0.143 |
| Map sharpening B factor (Å$^2$) | |
| Protein | 56.48 |
| Ligand | 41.41 |
| **Model composition** | |
| Non-hydrogen atoms | 1850 |
| Proteins residues | 250 |
| Ligands | 10 |
| B factors (Å$^2$) | |
| **R.m.s.d.** | |
| Bond lengths (Å) | 0.005 |
| Bond angles (°) | 0.704 |
| **Validation** | |
| MolProbity score | 1.91 |
| Clashscore | |
| 26.55 Rotamers outlier (%) | 0 |
| **Ramachandran plot** | |
| Favored (%) | 100 |
| Allowed (%) | 0 |
| Disallowed (%) | 0 |

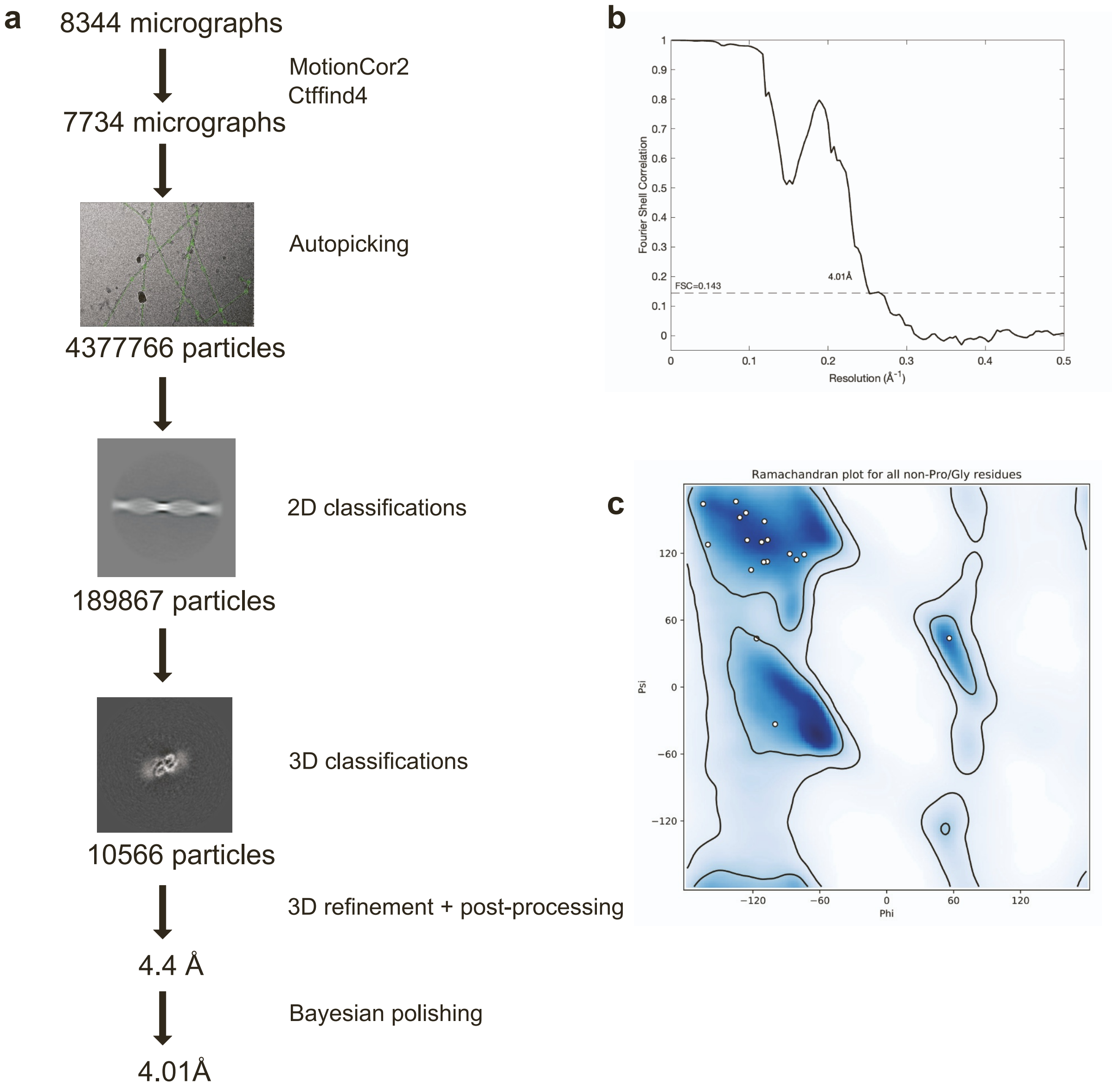


Figure S1: a) Flowchart for cryo-EM data analysis for the dominant polymorph of hIAPP-WT (PDB: 8R4I). b) FSC curves between two half-maps. c) Ramachandran plot obtained from Phenix.

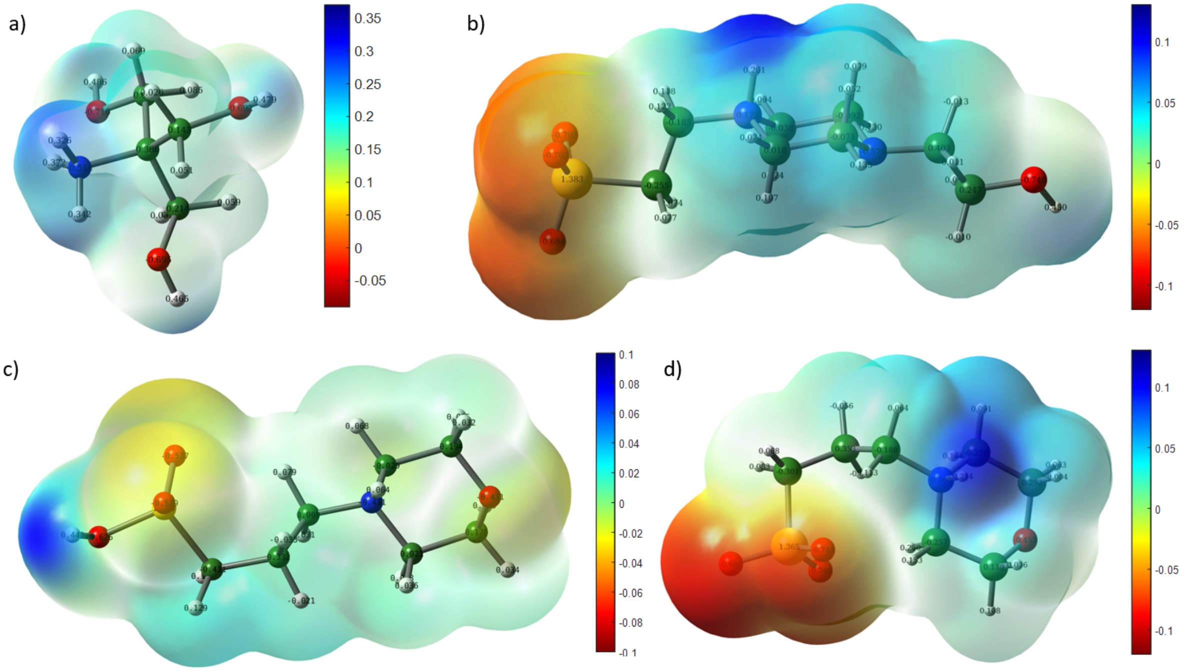


Figure S2: Electrostatic potential maps of a) Tris-HCl in its protonated form, b) HEPES in its zwitterionic form, c) MOPS in its neutral form and d) MOPS in its zwitterionic form.

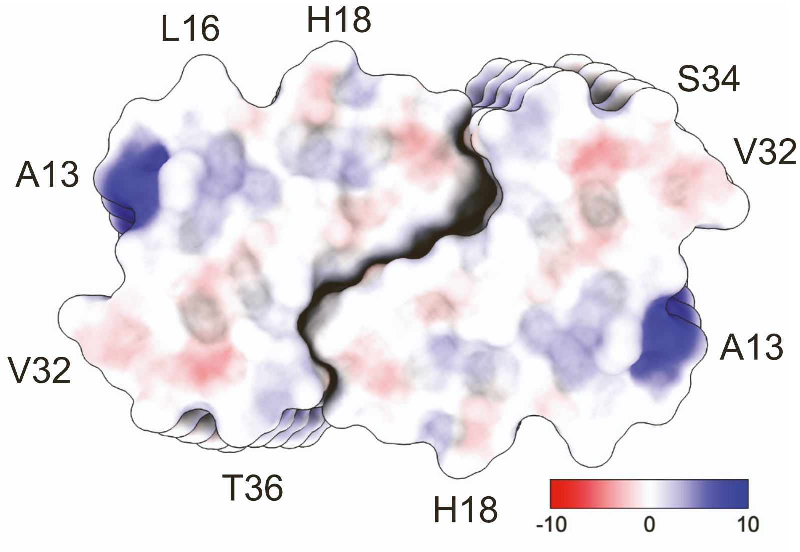


Figure S3: The electrostatic potential map of the solved WT-hIAPP structure. The map was generated with ChimeraX(8).

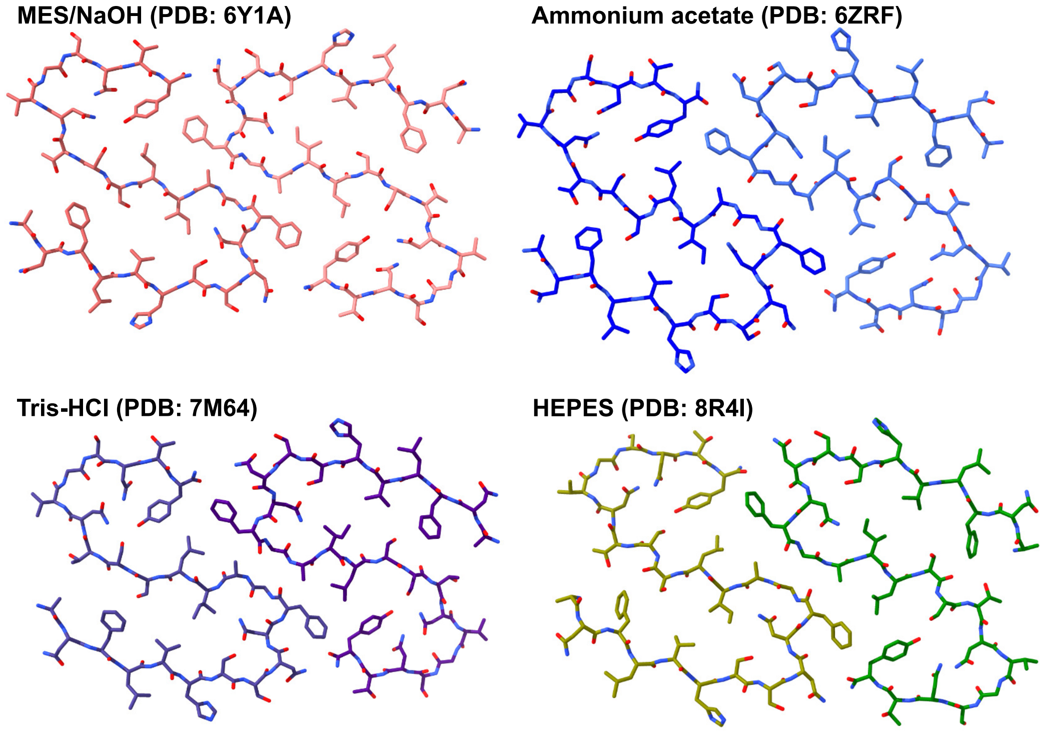


Figure S4: The dominant polymorph of hIAPP-WT aggregated in various buffer conditions. All the structures have almost identical backbone arrangements with exceptions in some rotameric amino acids.

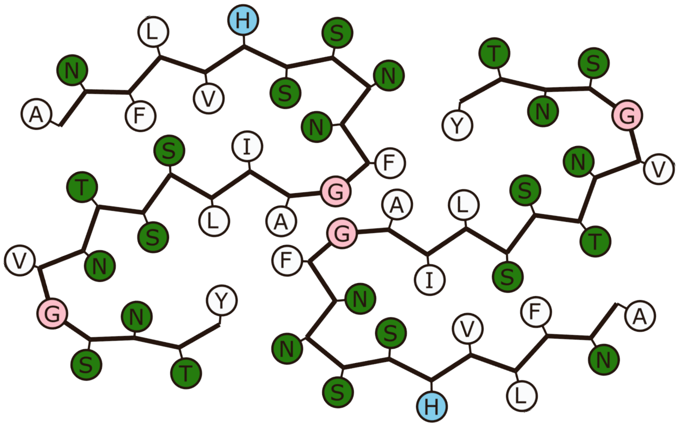

Figure S5: Schematic representation of hIAPP-WT, produced using atom2svg.py(9). The amino acids are color-coded according to their chemical properties. Positively charged residues are colored blue, polar residues are colored green, non-polar residues are colored white, and glycines are colored pink.

## REFERENCES


1. Scheres, S. H., 2012. RELION: implementation of a Bayesian approach to cryo-EM structure determination. *J. Struct. Biol.* 180:519–530.

2. Zheng, S. Q., E. Palovcak, J.-P. Armache, K. A. Verba, Y. Cheng, and D. A. Agard, 2017. MotionCor2: anisotropic correction of beam-induced motion for improved cryo-electron microscopy. *Nat. Methods* 14:331–332.

3. Rohou, A., and N. Grigorieff, 2015. CTFFIND4: Fast and accurate defocus estimation from electron micrographs. *J. Struct. Biol.* 192:216–221.

4. Bepler, T., A. Morin, M. Rapp, J. Brasch, L. Shapiro, A. J. Noble, and B. Berger, 2019. Positive-unlabeled convolutional neural networks for particle picking in cryo-electron micrographs. *Nat. Methods* 16:1153–1160.

5. Emsley, P., and K. Cowtan, 2004. Coot: model-building tools for molecular graphics. *Acta Cryst. D* 60:2126–2132.

6. Liebschner, D., P. V. Afonine, M. L. Baker, G. Bunkóczi, V. B. Chen, T. I. Croll, B. Hintze, L.-W. Hung, S. Jain, A. J. McCoy, et al., 2019. Macromolecular structure determination using X-rays, neutrons and electrons: recent developments in Phenix. *Acta Cryst. D* 75:861–877.

7. Pettersen, E. F., T. D. Goddard, C. C. Huang, E. C. Meng, G. S. Couch, T. I. Croll, J. H. Morris, and T. E. Ferrin, 2021. UCSF ChimeraX: Structure visualization for researchers, educators, and developers. *Protein Sci.* 30:70–82.

8. Meng, E. C., T. D. Goddard, E. F. Pettersen, G. S. Couch, Z. J. Pearson, J. H. Morris, and T. E. Ferrin, 2023. UCSF ChimeraX: Tools for structure building and analysis. *Protein Sci.* 32:e4792.

9. Nakane, T., version 210224. atom2svg. https://gist.github.com/biochem-fan/026ec2f191fee9285424d12fc2b84ce7.
