# Supplementary material for: Improving cryo-EM grids for amyloid fibrils using interface-active solutions and spectator proteins
Source: Biophys J. 2024 Feb 17;123(6):718–29. doi: 10.1016/j.bpj.2024.02.009 (PMC10995402; doi:10.1016/j.bpj.2024.02.009)
Supplement: Document S2. Article plus supporting material [file mmc2.pdf]

Biophysical Journal
Article

Biophysical Society

# Improving cryo-EM grids for amyloid fibrils using interface-active solutions and spectator proteins

Dylan Valli,[1] Saik Ann Ooi,[2] Giorgio Scattolini,[1] Himanshu Chaudhary,[1] Alesia A. Tietze,[2] and Michał Maj[1,*]
[1]Department of Chemistry – Ångström Laboratory, Uppsala University, Uppsala, Sweden and [2]Department of Chemistry and Molecular Biology, University of Gothenburg, Gothenburg, Sweden

ABSTRACT Preparation of cryoelectron microscopy (cryo-EM) grids for imaging of amyloid fibrils is notoriously challenging. The human islet amyloid polypeptide (hIAPP) serves as a notable example, as the majority of reported structures have relied on the use of nonphysiological pH buffers, N-terminal tags, and seeding. This highlights the need for more efficient, reproducible methodologies that can elucidate amyloid fibril structures formed under diverse conditions. In this work, we demonstrate that the distribution of fibrils on cryo-EM grids is predominantly determined by the solution composition, which is critical for the stability of thin vitreous ice films. We discover that, among physiological pH buffers, HEPES uniquely enhances the distribution of fibrils on cryo-EM grids and improves the stability of ice layers. This improvement is attributed to direct interactions between HEPES molecules and hIAPP, effectively minimizing the tendency of hIAPP to form dense clusters in solutions and preventing ice nucleation. Furthermore, we provide additional support for the idea that denatured protein monolayers forming at the interface are also capable of eliciting a surfactant-like effect, leading to improved particle coverage. This phenomenon is illustrated by the addition of nonamyloidogenic rat IAPP (rIAPP) to a solution of preaggregated hIAPP just before the freezing process. The resultant grids, supplemented with this "spectator protein", exhibit notably enhanced coverage and improved ice quality. Unlike conventional surfactants, rIAPP is additionally capable of disentangling the dense clusters formed by hIAPP. By applying the proposed strategies, we have resolved the structure of the dominant hIAPP polymorph, formed in vitro at pH 7.4, to a final resolution of 4 Å. The advances in grid preparation presented in this work hold significant promise for enabling structural determination of amyloid proteins which are particularly resistant to conventional grid preparation techniques.

SIGNIFICANCE This study presents a methodological advancement in the cryoelectron microscopy of amyloid fibrils. It elucidates the role of buffers and small proteins in grid preparation, emphasizing how alterations in solution properties enhance ice quality and fibril distribution on cryoelectron microscopy grids. These insights offer a practical approach to facilitate the structural determination of amyloid proteins, enabling the exploration of structures formed under diverse aggregation conditions. The proposed methods potentially apply to amyloid structures derived directly from tissue samples, opening up new possibilities for approaching more challenging fibril samples in future structural studies.

## INTRODUCTION

Cryoelectron microscopy (cryo-EM) has revolutionized the structural study of amyloid proteins owing to its exceptional ability to resolve high-resolution structures of individual fibril polymorphs, even within mixtures of diverse fibrillar species and in complex solution environments (1). As one of the most rapidly evolving technologies in the field, cryo-EM has seen tremendous advances in optics, detection systems, data acquisition methods, data processing algorithms, and innovative machine learning applications (2,3). Resolutions as high as 1.15 Å have been reported for apoferritin protein (4), whose structure was derived from single-particle analysis of cryo-EM micrographs. Helical reconstruction techniques have enabled solving the structure of tau amyloid protein to 1.86 Å resolution (5). Despite these tremendous successes, solving 3D structures of amyloid proteins remains a significant challenge, largely due to the unpredictable and error-prone grid preparation process. Grids for cryo-EM imaging are prepared through a process known as vitrification (6). Initially, a sample is applied onto a grid, and then any

*Submitted December 4, 2023, and accepted for publication February 13, 2024.*

*Correspondence: michal.maj@kemi.uu.se

Dylan Valli and Saik Ann Ooi contributed equally to this work.

Editor: Bridget Carragher.

https://doi.org/10.1016/j.bpj.2024.02.009

© 2024 Biophysical Society.

This is an open access article under the CC BY license (http://creativecommons.org/licenses/by/4.0/).

excess is removed using filter paper before the grid is rapidly plunge-frozen in ethane to form a thin layer suitable for imaging. Commercial instruments such as Vitrobot are often employed to ensure reproducible grid preparation by stabilizing various parameters, such as humidity, temperature, blotting force, and blotting time (7). Despite this controlled environment, the quality of the prepared grids may still be inconsistent. Consequently, low reproducibility of ice thickness is common, even under identical preparation conditions. In addition, controlling environmental contamination, particularly from high humidity poses a significant challenge (8,9). Compared with other proteins, amyloid proteins are frequently more difficult to deposit uniformly on grids. This is partly due to their elongated, fibrillar nature, as fibrils can be easily stripped off during the blotting process, leading to the inconsistency of fibril concentration on the grid (10). Moreover, fibrils tend to associate into dense, highly bundled clusters that are not suited for any high-resolution structure determination. The formation of individual, evenly dispersed fibrils is further impeded by their tendency to adhere to the grid materials, such as carbon, rather than remaining suspended in the vitreous ice solution. To mitigate these issues, surfactants are often introduced before grid preparation (11). Such an approach is particularly crucial when studying membrane proteins and other biomolecules that are prone to adopting a preferred orientation due to interactions at the air-water interface (12,13). For instance, in membrane protein studies, detergents are used to simulate bilayer environments (14), whereas the addition of detergents to soluble proteins can diminish preferred orientation (15), thereby facilitating the visualization of multiple orientations in collected micrographs. Although detergents are useful in grid preparation, especially in stabilizing membrane proteins, they often need to be present in relatively large quantities to exceed the critical micelle concentration (CMC) (16). For high CMCs, the presence of detergent in the samples can reduce the contrast between the protein and the background (17). Furthermore, the use of detergents does not guarantee that the preferred orientation of proteins does not occur. The use of other surfactants, such as amphipathic polymers (18,19), nanodiscs (20), or combination of amphipathic polymers and low-CMC surfactants (21) has instead become an alternative to conventional detergents. Besides reducing preferred orientation, the use of surfactants is also known to improve ice thickness and prevent particle aggregation near the edges of grid holes (21). However, it is not known yet if such an approach works well for amyloid proteins. The advantage of working with amyloid fibrils lies in their extreme stability and resistance to disaggregation, which allows for the application of various solution additives without the risk of compromising their structural integrity. However, some care needs to be exercised when selecting the right surfactant as some studies have indicated that the presence of strong surfactants, such as sodium dodecyl sulfate (SDS), might disrupt the secondary structure of shorter fibrils or protofilaments (22,23). In this study, we outline critical insights from hundreds of grid optimization efforts to uniformly distribute helically twisted amyloid fibrils formed by the human islet amyloid polypeptide (hIAPP) at physiological pH. hIAPP is a hormone peptide associated with the $\beta$ cell loss in type 2 diabetes. It exhibits diverse polymorphism and has been a particularly challenging study case for both solid-state NMR and cryo-EM (24,25). Our results demonstrate that the distribution of fibrils and the integrity of the grids are dictated by the properties of the solution during the final step of grid preparation rather than the initial growth conditions, such as buffers and salts. The presence of either accompanying proteins or low concentrations of detergents provided a tremendous improvement on the grids. These insights enable us to recommend optimal buffers for fibril imaging and establish comprehensive strategies for effective grid preparation that are adaptable to different amyloid proteins, pH levels, and are independent of the initial fibril growth conditions. The results offer a clear path to higher-quality imaging and a deeper understanding of key structures in amyloid-associated diseases.

## MATERIALS AND METHODS

All chemicals were purchased from Sigma-Aldrich, Taufkirchen, Germany, unless otherwise specified.

### Peptide synthesis and purification

The peptides were synthesized on a 0.10 mmol scale using 9-fluorenylmethyloxy-carbonyl (Fmoc) chemistry with an Intavis MultiPep CF peptide synthesizer (Tübingen,Germany). Tentagel R RAM resin (0.21 mmol/equiv) was used to produce a C-terminal amide. Pseudoprolines derivatives were used to prevent aggregation and improve coupling during synthesis (26). Fmoc-Ala-Thr(Psi(Me,Me)pro)-OH was used at positions 8 and 9 and Fmoc-Leu-Ser(Psi(Me,Me)pro)-OH at positions 27 and 28. A trifluoroacetic acid-based cleavage cocktail (95% trifluoroacetic acid, 2.5% triisopropylsilane, 2.5% $H_2O$) was used to cleave the synthesized peptides. Crude peptides were dissolved in $H_2O$ and lyophilized. The peptides were then dissolved in DMSO mixed with 25% acetic acid solution at a 60:40 ratio, and incubated at 500 rpm at room temperature for 24 h to form the disulfide bridge. Peptides were purified using reverse-phase HPLC (Isera C18 preparative column, 250 × 20 mm, Düren, Germany), with a gradient elution composed of buffer A (100% $H_2O$ with 0.045% HCl) and buffer B (80% acetonitrile with 0.045% HCl). The method used was 0%B to 40%B in the first 15 min, thereafter 40%B to 50%B from 15 to 30 min. The peptides usually eluted around 45%B and they were collected and lyophilized overnight. Second purification was carried out by dissolving the peptide in 50% hexafluoroisopropanol and 20% acetic acid. The eluted peptides were lyophilized overnight. Liquid chromatography-mass spectrometry was used to confirm the mass of the synthesized peptides. The purified peptides were dissolved in hexafluoroisopropanol for several hours. A 10 $\mu$L of stock was evaporated with nitrogen gas and resuspended in $H_2O$ to determine the concentration using absorbance at A280 nm with extinction coefficient of 1490 $M^{-1}$ $cm^{-1}$. The peptides were aliquoted based on the concentration needed and were lyophilized overnight and stored at −20°C until use.

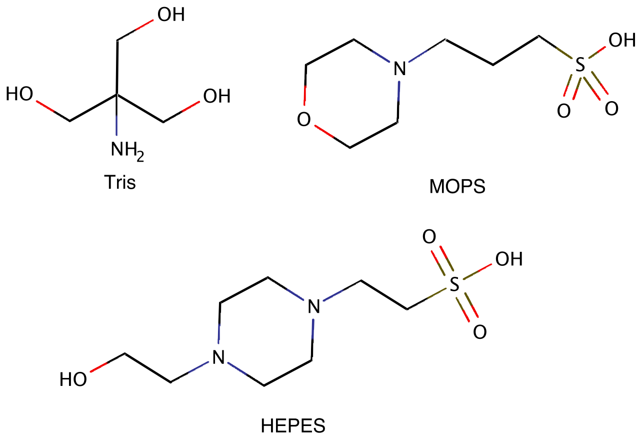


FIGURE 1 Molecular structures of Tris, MOPS, and HEPES buffers used in the study. To see this figure in color, go online.

## Peptide aggregation conditions

Amyloid fibrils were formed from the monomeric hIAPP under varying buffer conditions. 2-[4-(2- hydroxyethyl)piperazin-1-yl] ethanesulfonic acid (HEPES) (Fisher Bioreagent, Pittsburgh, PA, USA) and tris(hydroxymethyl)aminomethane (Tris) (Fisher Bioreagent) were selected to elucidate the fibril formation and grid quality at physiological pH, whereas 3-morpholinopropane-1-sulfonic acid (MOPS) (PanReac AppliChem, Darmstadt, Germany) was chosen to test for both acidic and physiological pH with the use of the same buffering agent. The monomer wild-type (WT) was aggregated in either one of the buffers: 20 mM Tris-HCl at pH 7.4, MOPS at pH 6.5, MOPS at pH 7.4, 20 mM HEPES at pH 7.4 for 7 days at room temperature and quiescent condition. To ensure complete aggregation, the kinetics were monitored using a Thioflavin-T fluorescence assay following standard protocols.

## Grid preparations

Samples (3 $\mu$L) were added to the holey-carbon grids (Quantifoil R 3.5/1, 200 mesh, Cu, Großlöbichau, Germany), which were glow-discharged for 90 s at 20 mA. The grids were blotted for 3 s with blot force 0, at 4°C, 95% humidity, and plunge-frozen immediately into liquid ethane using Vitrobot Mark IV (Thermo Fisher Scientific).

## Cryo-EM data acquisition and processing

For screening, all samples were imaged on 200 kV Glacios (Thermo Fisher Scientific) equipped with a field emission gun. Images were collected at 510× magnification for the grid square view, 8500× magnification for the grid holes view, and 92,000× magnification for the acquisition view with a defocus between 2 and 4 $\mu$m. Images were collected on a 4 K × 4 K Falcon 3 Direct Electron Detector (Thermo Fisher Scientific,) with a pixel size of 1.6 Å/pixel using EPU software (Thermo Fisher Scientific). For data collection of WT in the HEPES buffer, the micrographs were collected on a 300 kV Titan Krios (Thermo Fisher Scientific) equipped with a field emission gun. Images were collected at 105,000 magnification with a defocus between 0.5 and 1.7 $\mu$m. Images were recorded on a 6 K × 4 K K3 BioQuantum electron detector (Gatan) with a pixel size of 0.828 Å/pixel using EPU software (Thermo Fisher Scientific). All data processing was performed using RELION 4.0 (27). Detailed data processing steps are provided in the supporting material.

## Surface tension measurements

The surface tension of Milli-Q water, HEPES, Tris-HCl, and SDS was measured using the Wilhelmy plate apparatus. A slide of silica glass (length 24.1 mm) was hung from a holder, positioned on top of an SE 203 LR scale (VWR). Before every measurement, the glass slide and the beaker containing the samples were washed first with 2.5 M NaOH 1:5 (v/v) $H_2O$:EtOH and then with 6 M HCl.

## Ab initio calculations

The electrostatic potential maps of Tris-HCl, MOPS, and HEPES were calculated with density functional theory using the B3LYP functional (28) and the 6-311+G(d,p) basis set (29–33). The calculations were performed with the Gaussian 16.C suite of packages (34). The computed electrostatic maps were visualized using GaussView 6.1.1 (35).

# RESULTS

## The influence of buffer composition

We formed amyloid fibrils from monomeric hIAPP under various buffer conditions. Tris-HCl, HEPES, and MOPS were chosen to investigate the formation of fibrils and the quality of grids at physiological pH. The molecular structures of the studied buffers are shown in Fig. 1. Tris-HCl is a popular choice in many biochemical studies for its broad pH range and inert behavior in physiological environments. Numerous past studies of hIAPP aggregation have utilized Tris-HCl buffer at pH 7.4 and this prompted our selection for consistency and comparability with prior research (23,36,37). HEPES and MOPS buffers were chosen to explore whether larger organic buffer molecules affect fibril formation. MOPS was specifically chosen for its capability to have a buffering range that lies in both acidic and physiological pH levels. This choice was informed by a previously reported successful cryo-EM study that used 2-(N-morpholino)ethanesulfonic acid (MES)/NaOH buffer at pH 6 (38). Each grid in our study was assessed based on three key quality indicators: ice coverage uniformity, tendency of fibrils to form dense clusters, and the presence of evenly dispersed fibril filaments that are suitable for high-resolution structure determination. To provide a quantitative assessment of ice quality, we selected three atlases for each sample, from which three grid squares were randomly chosen. This approach yielded a total of nine grid squares per condition, which included approximately 1250 grid holes. We calculated the average ice coverage across these holes and normalized the values to express the percentage of ice coverage per grid square. In addition, we quantified the degree of fibril clustering by calculating the integrated area of dense fibril clusters across five atlases, equivalent to 125 grid squares, and then normalized this area per atlas. The results are compiled in Table 1.

The buffer screening results are shown in Fig. 2, which highlights the overall ice quality by displaying grid holes at low magnification and the distribution of fibrils within

**TABLE 1 Quantitative analysis of ice coverage and fibril clustering on cryo-EM grids under different conditions**

| Condition | Ice coverage per grid square (%) | Area covered by clumps ($\mu m^2$) |
|---|---|---|
| Tris-HCl | 21.3 ± 17.0 | 849 |
| HEPES | 78.5 ± 25.0 | 192 |
| Exchanged HEPES 50 mM | 37.4 ± 23.9 | 429 |
| SDS | **94.1 ± 6.3** | 495 |
| DDM | 28.3 ± 14.0 | 1089 |
| CTAC | 57.7 ± 14.4 | 1008 |
| rIAPP | 86.9 ± 8.1 | **14** |

Ice coverage is calculated as the percentage of suitable grid holes, based on ice thickness and absence of contamination, averaged from nine grid squares across three atlases (approximately 1250 grid holes). Fibril clustering is measured as the percentage area of clumps, assumed to be circular, in approximately 25 grid squares per atlas. These values provide a standardized evaluation of grid quality and amyloid fibril distribution for each sample condition. Best conditions in each test (SDS has the best ice coverage, and rIAPP has the lowest [best] area covered by clumps) are highlighted in bold.

the grid holes at higher magnification. Each condition was tested at least three times to ensure that poor grid quality did not result from human error during the operation of the Vitrobot. Initially, we selected MOPS buffer at pH 6.5 to replicate the high grid quality reported with MES/NaOH by Röder et al. (38). At this stage, we had already made several unsuccessful attempts to prepare grids at physiological pH. Therefore, adopting conditions that had proven successful for other groups was our initial strategy, which set the stage for a gradual, stepwise progression toward achieving similar grid quality at pH 7.4. As shown in Fig. 2, the grids prepared with MOPS buffer at low pH exhibit excellent fibril coverage, with the majority of the long, well-separated fibrils displaying a helical twist. This finding aligns well with the previously reported data for MES buffer, even though the pH was 0.5 units higher in our study. Structurally, MES and MOPS differ by only a single methylene group separating the morpholine ring from the sulfonate headgroup, suggesting that similar interactions are present in both buffers. MOPS buffer is characterized by a pH range between 6.5 and 7.9. Therefore, in the subsequent step, we applied MOPS at the same concentration but adjusted to physiological pH. We have observed that the fibrils grown in MOPS buffer at pH 7.4 tended to clump severely, making the selection of individual fibril filaments for cryo-EM structure determination impossible. In addition, a greater concentration of condensed clusters was observed on the carbon grid material rather than in the thin layer of vitreous ice. This observation prompted us to test various types of grids. We selected Quantifoil R 3.5/1 instead of the standard 2/2 grid because its larger holes offer the additional surface area to support the fibrils and potentially minimize contact with the edges. While the 3.5/1 grid did not reduce clumping on the carbon, the greater area available for ice coverage led us to continue using this grid type in all of our amyloid studies. Preparing grids with Tris-HCl buffer proved even more challenging than with MOPS. In addition to clumping and accumulation at the hole edges, the overall ice quality deteriorated, and controlling and reproducing the ice thickness became more difficult. On average, only 21% of grid holes contained appropriate ice layers. We evaluated additional neutral pH buffers (PBS, PIPES) and observed outcomes comparable

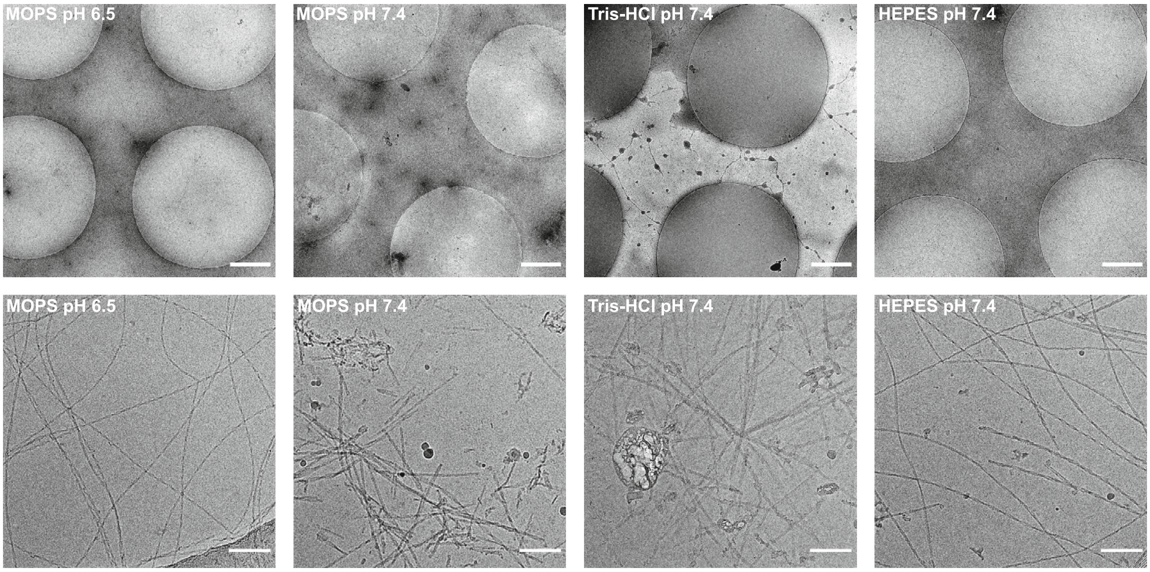


FIGURE 2 Cryo-EM micrographs showing hIAPP fibrils aggregated under different buffer conditions. The top panel presents micrographs at grid holes magnification (8500×) while the bottom panel shows micrographs at acquisition magnification (92,000×). Among all the tested physiological pH buffers, only HEPES buffer yields good ice coverage and a satisfactory distribution of fibrils. While MOPS buffer did not facilitate adequate fibril coverage, lowering the pH to a more acidic level significantly improved both fibril coverage and the ice quality. Scale bars, 1 $\mu$m (grid holes) and 100 nm (acquisition scale bar).

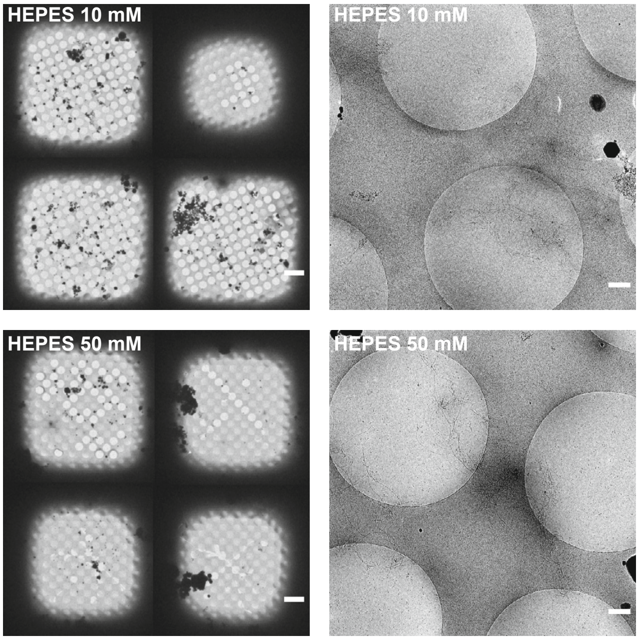


FIGURE 3 Cryo-EM micrographs showing hIAPP aggregated in Tris-HCl followed by buffer exchange to HEPES at final concentrations of 10 and 50 mM. Micrographs were collected at grid square magnification (512×, *left*) and grid hole magnification (8500×, *right*). The grid square view reveals a trend shift from predominantly dry holes at low HEPES concentration to a majority of holes with a well-formed ice layer. In both scenarios, clumps of fibrils are still observable. Scale bars, 10 $\mu$m (grid squares) and 500 nm (grid holes).

with those already described, so these findings have been excluded from the current study. The exception is the HEPES buffer, which we will examine in more detail in the subsequent discussion. Among all the conditions studied, grids prepared in HEPES buffer show significantly better coverage and ice quality. The ice coverage increases from 21.3% in Tris-HCl to 78.5% in HEPES. It is also the only physiological pH buffer where substantial clumping is not observed, allowing individual fibrils to be easily distinguished and selected during data processing. We observe approximately four times less area covered by dense fibril clusters compared with Tris-HCl. While there is still a tendency for fibrils to associate near the grid edges, this effect is much less pronounced compared with other buffers, resulting in a higher proportion of uniformly distributed fibrils within the ice layer. This finding motivated us to conduct further investigation into the physical origins of such improvement in ice control. Structurally, HEPES shares the sulfonate headgroup with many buffers tested in this study, and its piperazine ring is also a feature of PIPES, which did not yield similarly positive results. The distinctive structural element of HEPES is the free hydroxyl group situated at the end of the piperazine ring, opposite the sulfonate. To elucidate the physical mechanisms at play, we conducted buffer exchange experiments and hydrodynamic tests to determine whether fibrils grown under various conditions could be suitably imaged when HEPES or other molecules were introduced into the solution as additives. This would allow us to determine if the improved grid quality is a consequence of the specific protein interactions with the buffer components or if it arises from the stabilization of the thin ice layers due to alterations in the microscopic behavior of the aqueous solution in the blotting process.

## Buffer exchange and effects of solution additives on ice quality and fibril distribution

To deliver a deeper understanding of the impact of buffer composition on the quality of cryo-EM grids, we performed a series of buffer exchange experiments, in which fibrils initially aggregated in Tris-HCl were subjected to the addition of HEPES buffer at varying concentrations before freezing. The final concentrations of HEPES were 10 and 50 mM, respectively. In the data presented in Fig. 3, a concentration-dependent improvement in ice coverage was observed, with the 50 mM concentration demonstrating the most notable enhancement. The ice layers formed under this condition showed twofold improvement in the ice coverage per grid square. However, despite the improved ice quality, the distribution of fibrils remained unsatisfactory. Most fibrils were predominantly located on the carbon support rather than being evenly dispersed within the grid holes. This observation suggests that, while HEPES buffer aids in forming stable ice, it alone is not sufficient to ensure a uniform distribution of particles observed in the case where the peptide was aggregated in HEPES buffer from the start. The improvement in ice quality upon the addition of HEPES led us to suspect that constituents of certain buffers may have a direct effect on the ice formation process by affecting the behavior of proteins in the vicinity of the air-water interface, potentially mimicking a surfactant-like effect. To test if the surfactant monolayers improve the overall quality of grids containing amyloid fibrils, we conducted experiments using a range of surfactants: anionic SDS, nonionic n-dodecyl $\beta$-D-maltoside (DDM), and cationic cetyltrimethylammonium chloride (CTAC). Our objective was to assess their respective effects on the quality of grids containing amyloid fibrils. All surfactants were used below their CMC. Interestingly, the addition of SDS results in the most stable ice observed in all our experiments, as shown in Fig. 4. The majority of grid holes were filled with ice of nearly identical thickness, indicating a robust stabilizing effect of SDS on vitreous ice film formation, with 94.1% grid holes containing high quality ice. However, the distribution of fibrils showed only a slight improvement, with elongated fibrils more frequently entering the grid holes, but still clustering on the carbon. This suggests that, while SDS effectively stabilizes the ice layer, achieving an ideal distribution of amyloid fibrils for cryo-EM analysis requires a combination of surfactants and other additives designed to enhance the separation between fibril filaments. In contrast,

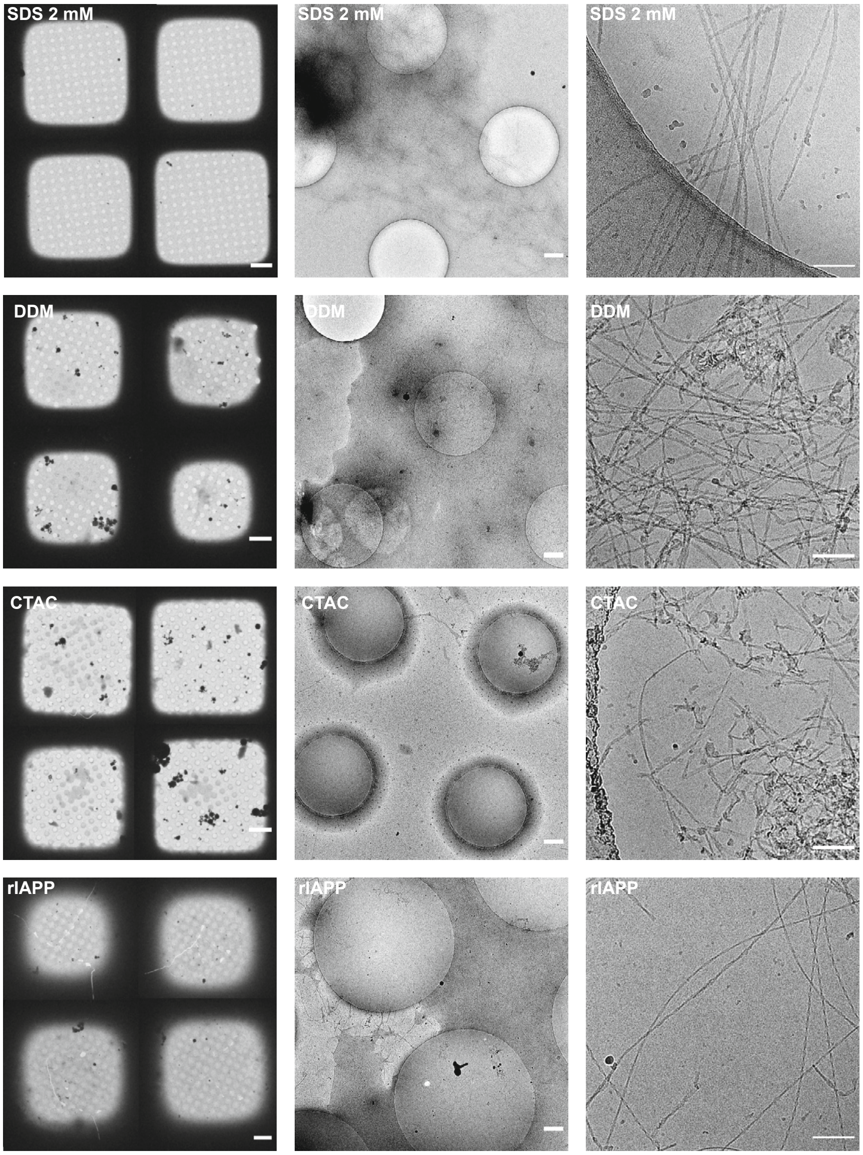


FIGURE 4 Cryo-EM micrographs of hIAPP aggregated in Tris-HCl with the addition of SDS, n-dodecyl-$\beta$-D-maltoside (DDM), cetyltrimethylammonium chloride (CTAC), or rIAPP as additive before grid preparation. Micrographs were collected at grid squares magnification (512×, *left*), grid holes magnification (8500×, *middle*), and data acquisition magnification (92,000×, *right*). The presence of 2 mM SDS led to a thin ice layer in every hole, improving the overall quality of the grid greatly. DDM and CTAC also led to better ice quality but to a lesser extent than SDS, with large fibril clumps still visible. The addition of rIAPP not only improved the ice layer but also showed a better distribution of fibrils and less clumping. Scale bars, 10 $\mu$m (grid square), 500 nm (grid holes), and 100 nm (acquisition scale bar).

DDM showed no improvement in ice quality compared with the pure Tris-HCl buffer. This outcome suggests its limited effectiveness in this context or points to the need for precise tuning of its concentration to achieve the desired effects. CTAC, on the other hand, demonstrated some effectiveness with about 57.7% ice coverage, indicating a twofold improvement over Tris-HCl. However, it is important to note that both DDM and CTAC led to a substantial increase in areas with clumped fibrils, surpassing even those observed with the buffer alone. These findings suggest that, while SDS significantly enhances ice layer stability, the other surfactants tested, DDM and CTAC, do not offer similar benefits and in fact may exacerbate fibril clumping issues. Therefore, achieving optimal fibril distribution for cryo-EM analysis might require a more tailored approach, potentially involving a carefully selected combination of surfactants and other additives to effectively separate fibril filaments.

The formation of stabilizing monolayers at the interface, besides conventional surfactants, has also been reported for denatured proteins. To shed more light on such a possibility, we examined the impact of adding small amounts of nonamyloidogenic rat IAPP to the hIAPP solution before grid freezing. The final concentrations of rat IAPP (rIAPP) and hIAPP were 50 and 25 $\mu$M, respectively. Grids prepared with rIAPP addition showed a significant enhancement in ice quality, achieving 86.9% coverage of ice and a negligible amount of clustered areas. Although the ice film was not as uniform as that formed in the presence of SDS, as illustrated in Fig. 4, the improvement was still remarkable. The results suggest that rIAPP might form a monolayer at the air-water interface consistent with previously proposed mechanisms,

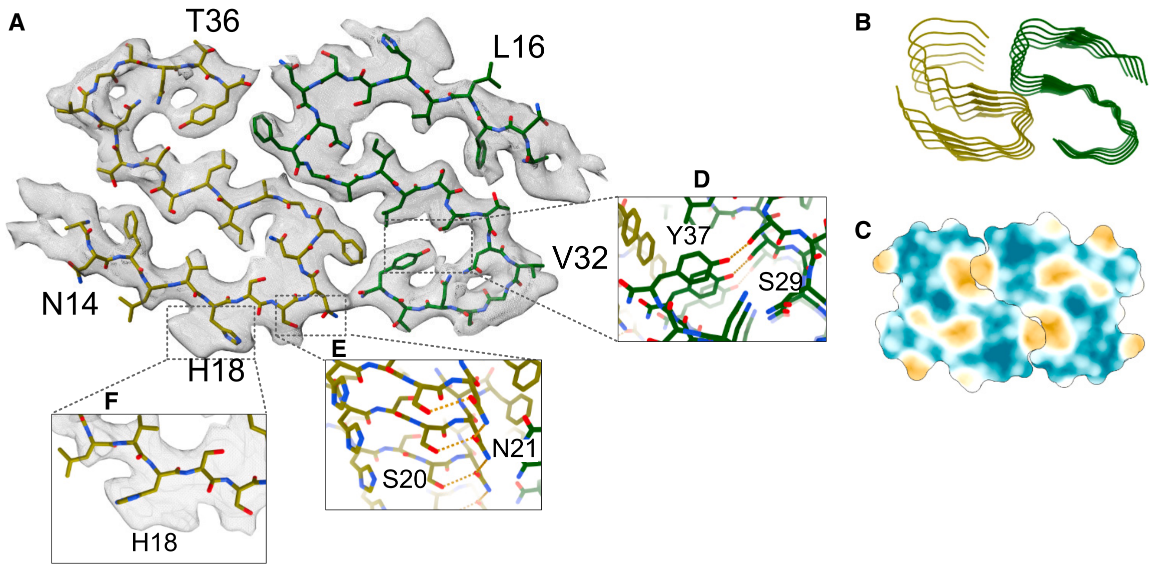


FIGURE 5 (*A*) Density map showing the structure of the predominant polymorph found in hIAPP fibrils aggregated in HEPES (pH 7.4). The N-terminal residues 1–12 are unresolved, reflecting their high flexibility. (*B*) Illustration of lateral stacking in peptide units with $\beta$ sheets depicted as ribbons. (*C*) Hydrophobicity map highlighting the interface between individual protofilaments, with hydrophobic interactions in yellow and hydrophilic in teal. (*D*) Detailed view of the stabilizing hydrogen bonds between Tyr37 and Ser29. (*E*) Representation of Asp21 forming ladder-type interactions, stabilizing the stacking of peptide units. (*F*) Close-up of the density around His18, suggesting the presence of two rotameric conformations at this site. To see this figure in color, go online.

consequently stabilizing the ice layer. Most importantly, besides improving ice films, rIAPP seems to facilitate more uniform dispersion of fibrils within the grid holes, reducing the tendency of fibrils to adhere to the carbon support. It appears that the presence of the nonaggregating spectator protein may induce intermolecular interactions between monomeric rIAPP and hIAPP fibrils. This, in turn, may lead to improvement in separation between individual filaments, further contributing to the enhanced dispersion and stabilization of fibrils on the cryo-EM grids. It is also possible that rIAPP competes with fibrillar species for binding sites on the amorphous carbon film. This competition may decrease the fibrils' affinity for the carbon, leading to an increased number of fibrils entering the grid holes.

## Cryo-EM structure determination

The enhanced ice coverage and the abundance of monodisperse fibril filaments observed with HEPES buffer facilitated the determination of fibril structures formed at physiological pH. The cryo-EM data processing workflow is shown in detail in Fig. S1, and the final structure fitted to the refined map is depicted in Fig. 5. Initially, 8344 micrographs were collected, motion-corrected with MotionCor2 (39), and underwent contrast transfer function estimation using CTFFIND4 (40). Autopicking using Topaz (41) yielded over 4.3 million particles. The 2D classification step enabled us to discard improperly picked areas resulting from the low threshold used in the autopicking algorithm and to exclude unusable fibril particles due to either poor resolution or absence of helical symmetry. Notably, over 80% of the particles corresponded to the same polymorph, with only a minor fraction representing polymorphs with longer crossover distances. Following several iterations, 189,867 particles of the dominant polymorph were selected for further refinement through the 3D classification process. This classification refined the particle selection, resulting in 10,566 particles that advanced to the final 3D refinement and postprocessing stages. The resolution of the reconstructed 3D structure after Bayesian polishing was determined to be 4.01 Å based on the Fourier shell correlation at the 0.143 criterion. The final map presented clear density for the majority of the peptide, which enabled the construction of an accurate model. The structure is consistent with those previously reported, indicating that this particular polymorph can form under a wide range of conditions.

## DISCUSSION

The successful preparation of thin ice specimens in cryo-EM is of utmost importance, as it directly impacts the ability to resolve high-resolution 3D structures, a key objective in structural biology. This becomes particularly important in the preparation of amyloid samples, where the challenge lies not only in obtaining ice layers of the desired thickness but also in ensuring their overall stability against the mechanical stress imposed by elongated fibrillar species. Our results suggest that the stability of ice can be improved through various solution-altering approaches. However, stable ice layers alone are not sufficient for effective cryo-EM

analysis of fibrillar species, as their tendency to associate either with themselves or with support materials presents a significant challenge. Although careful selection of media used for the aggregation process may improve the distribution of filaments, our ultimate goal is to develop protocols that enable solving 3D structures of amyloid fibrils grown under any conditions, including samples directly extracted from human tissues. In the discussion that follows, we summarize potential approaches that may bring us closer to this goal and discuss the possible physicochemical explanations for their effectiveness.

## Factors contributing to stable ice formation

The factors influencing the formation and stability of thin vitreous ice films have been thoroughly examined by Glaeser et al. (42,43). They highlight that uniformly thin films of pure water are inherently unstable due to molecular interactions within the film. This instability often results in dewetting and rupture of the ice film. Crucially, the addition of surfactants to water can mitigate these fluctuations, thereby stabilizing the film. Such stabilization is achieved as surfactants form a monolayer at the air-water interface, reducing surface tension fluctuations and altering the disjoining pressure. In our study, the grids prepared with the addition of SDS solution supported this hypothesis. In the case of buffering agents, while some exhibited potential for stabilizing ice layers, the effects seem to arise from a more complex interplay, involving both potential stabilization of the air-water interface and direct interactions with the fibrillar protein samples. It has been suggested that HEPES could act as a surfactant (44). However, our surface tension measurements of 20 mM Tris-HCl and 100 mM HEPES show very small changes compared with the values of Milli-Q water (Table S1), which can be mostly attributed to changes in the solution density due to the high concentrations of solute. Moreover, the two buffers display statistically identical values of surface tension. Hence, it is not possible to simply explain the significant difference between the samples in HEPES and in Tris-HCl purely based on a surface tension argument. Therefore, the reason behind the different quality of ice is probably related to specific interactions of the buffering agents with the proteins. First, the electrostatic charge of the buffers at pH 7.4 is different: Tris-HCl is mostly in its protonated positively charged form, while the sulfonate groups of HEPES and MOPS are either in their deprotonated negatively charged forms or protonated neutral forms (45,46). Considering that the isoelectric point of hIAPP has been reported to be 9.6 (47), at pH 7.4 the fibrils are positively charged. Hence, it is plausible to assume that interactions with Tris-HCl, compared with HEPES and MOPS, are partially inhibited by electrostatic repulsion. To aid our understanding of the electrostatic forces at play, we carried out ab initio calculations of the studied buffers in their relevant protonation states. The electrostatic potential maps of the three buffering agents (Fig. S2) show that MOPS and HEPES at pH 7.4 display regions of negative and positive charge of similar magnitude, given their zwitterionic nature, while Tris-HCl has a predominantly positive charge that dominates the entire structure. Hence, the first two buffers can take part in a broader range of interactions than the latter. It is also important to notice that MOPS at pH 6.5 is 90% in its neutral form, while at pH 7.4 this value goes down to 30% (46). From the electrostatic potential maps of two distinct protonation states of MOPS (Fig. S2) it is possible to see a dramatic change in charge density that would lead to different interactions between the buffer and the protein. Hence, this could be the possible origin of the different behavior of the samples prepared in MOPS at different pH. Secondly, while both MOPS and HEPES can both participate in hydrogen bonding via the sulfonate and amine groups (48), HEPES can be involved in more hydrogen bonding interactions with its terminal hydroxyl group. Such interaction has already been observed via x-ray crystallography in multiple protein structures (49,50). Given the striking differences between the samples prepared in HEPES and the ones prepared in MOPS, the terminal hydroxyl group seems to have a crucial role in the buffer-peptide interactions that ultimately lead to the formation of fewer clumps and overall improved ice. Such differences between buffers and the consequential effects on the study of biomolecules are known and they have been extensively investigated (51). In particular, in the case of amyloid fibrils, it has been demonstrated by Mao et al. that HEPES and Tris-HCl have different effects on the aggregation of $hIAPP_{11–20}$ (52). One possible reason for the poorer quality of the ice in the samples presenting clumps is the potential ice nucleation activity of the aggregates. In particular, the hIAPP sequence presents the TxT motif (where T is threonine and x is a non-conserved amino acid) that is common in ice-nucleating and antifreeze proteins (53,54). It has been also demonstrated that aggregation of such proteins makes them more keen to induce ice nucleation rather than preventing it (55,56). Moreover, it has been shown that, even in the absence of the TxT motif, protein aggregation is able to induce ice nucleation (57). Hence, the fibril clusters present on the grid can act more efficiently as nucleation sites compared with the isolated fibrils. This could explain why the samples containing Tris-HCl present an ice layer of poorer quality compared with the ones containing HEPES. The electrostatic potential map of the protein is shown in Fig. S3. The map highlights that the most positive charge is located closer to the N-terminus. This is due to the presence of positively charged lysine and arginine residues and is consistent with the membrane interaction studies showing that hIAPP associates with a negatively charged membrane through its N-terminus (58). This means that, close to the potential ice nucleation site, there are positive charges that can repel Tris-HCl. In addition, HEPES improves the quality of ice,

possibly because of specific interactions with the ice nucleation sites. This would explain why the quality of the ice improves after exchanging the buffers, despite the fibrils distribution being unaffected. As previously mentioned, the effects of detergents on the preparation of cryo-EM grids have been already investigated, especially when it comes to the study of membrane proteins (11,59,60). In addition to stabilizing the air-water interface, detergents play a crucial role in stabilizing membrane proteins themselves, making their use quite common in that field. However, the implications of using a detergent during the preparation of amyloid fibrils samples for cryo-EM seem to have never been taken into consideration before. More interesting is the effect that rIAPP has on ice. It has been previously showed by Engel et al. that this protein can act as a surfactant (58). Being a disordered protein, presenting both polar and apolar domains, it is not surprising that it displays a surfactant behavior. Given its properties, it can be expected that rIAPP displays similar properties to amphipol polymers, such as A8-35, that can act as surfactants (61), and that are known to improve the quality of the ice layer (11).

## Disentangling the fibrils with spectator proteins

The addition of monomeric rIAPP to the solution of fully aggregated hIAPP fibrils offered unique insights into the intermolecular dynamics of amyloid structures. The primary goal of this approach was to establish if rIAPP could act as a surfactant and enhance the quality of ice on cryo-EM grids. Surprisingly, the grids frozen after the addition of rIAPP showed the most impressive grid coverage and fibril distribution compared with all the studied samples. This improvement was consistent, irrespective of the buffer used in the initial amyloid fibril formation process. Although HEPES buffer and SDS were found to improve the quality of ice, they did not reduce the formation of superstructures. Therefore, the observed improvement upon adding rIAPP is likely due to specific intermolecular interactions between hIAPP fibrils and the monomeric rIAPP, rather than general surfactant effects on the ice quality. Interactions between rIAPP and prefibrillar assemblies of hIAPP were previously studied with molecular dynamics simulations to evaluate the energetics associated with the formation of hybrid hIAPP-rIAPP fibrils (62). The interaction energies were evaluated across different parts of the peptide sequence, showing that the N-terminal side of rat IAPP, involving residues 10 to 18, exhibits the strongest stabilizing interaction energy when in direct interaction with hIAPP. This implies that there are high attractive forces driving the rIAPP molecules to interact with preformed or fully formed hIAPP fibrils. It must be noted that the interaction occurs with the fully conserved N-terminal part of the peptide, leaving the highly repulsive C-terminus, containing proline residues, at the other end. This in turn may reduce the propensity of amyloid fibrils to associate with each other when rIAPP molecules are close to their surface. Cryo-EM results also undoubtedly show that the N-terminus of aggregated hIAPP is outside the amyloid core, and instead forms flanking regions that are exposed to potential interactions with monomeric rIAPP. It must be noted that many studies involving rIAPP and hIAPP mixtures primarily focus on the inhibitory properties of rIAPP or the mechanisms of cross-seeding and hybrid fibril formation (63,64). In our case, however, we are concerned with the behavior of rIAPP in the presence of mature fibrils, rather than prefibrillar forms of hIAPP, which had never been seriously considered. From our perspective, it was critical to ensure that the presence of rIAPP does not affect the final aggregation products produced in other media, such as Tris-HCl. The use of proteins as stabilizers and contrast-enhancing scaffolds in high-resolution cryo-EM is a rapidly developing area. Protein scaffolds, in single-particle analysis, are attached to smaller molecules to assist in determining their structure (65). The potential of introducing "microscope invisible" crowders, such as small molecules or polymers that subtly influence the sample environment, has not yet been fully explored. Nonetheless, our results using small proteins in imaging amyloids offer a novel perspective and provide significant insights into the dynamics of amyloid structures such as those formed by hIAPP. Spectator proteins may enhance cryo-EM grid quality by reducing unwanted interactions or stabilizing specimens. Therefore, incorporating these proteins, particularly for complex biological structures such as amyloid fibrils, could significantly enhance the capabilities and insights offered by cryo-EM. Currently, we are investigating the application of protein crowders and polymers, such as amphipols, in a broader context of elucidating their ability to reduce the self-association of fibril species in cryo-EM specimens.

## Resolving in vitro structure of hIAPP fibrils formed at physiological pH: Insights and future directions

Resolving high-resolution structures of protein fibrils associated with human diseases is extremely important for the development of innovative diagnostic and therapeutic approaches. The capability to elucidate amyloid structures that form under physiological conditions is critical for ensuring that their structural characteristics are identical to the debilitating amyloid forms found in human tissues. To date, the most successful approach has been based on seeding them monomeric hIAPP with fibrils extracted from islet cells, which has resulted in four distinct polymorphic structures of hIAPP (25). Nevertheless, the ability to resolve in vitro structures in varied conditions holds immense value, particularly when biological samples for seeding are not accessible, or if the seeding fails to generate cryo-EM grids of sufficient quality for structural analysis. By applying HEPES buffer for the aggregation process, we achieved

optimal fibril distribution and ice layers of sufficient quality to resolve the high-resolution structural model of hIAPP fibrils (presented in Fig. 5). The double S polymorph observed at pH 7.4 is consistent with previously reported structures from both ex vivo and in vitro studies conducted at pH 6 (38,66). Our initial data processing of the fibrils produced in Tris-HCl buffer suggests that incorporating rIAPP enables the acquisition of high-resolution particles, suitable for subsequent 3D reconstruction. While this might seem like an aggressive strategy, particularly risky for small protein samples in single-particle cryo-EM, it is important to recognize the remarkable stability of amyloid fibril structures. The introduction of rIAPP is unlikely to significantly alter the amyloid core. Typically, amyloid disaggregation involves the use of harsh fluorinated solvents and strong acids, a process that is often slow and inefficient (67). Hence, we advocate for the addition of small, "microscope-invisible" proteins to facilitate grid preparation. We anticipate that this approach, with its potential for generalization to other amyloid diseases, will become valuable in revealing 3D structures of amyloid fibrils, including those extracted directly from tissues or formed in the presence of phospholipid membranes.

## DATA AND CODE AVAILABILITY

Structural data have been deposited into the Worldwide Protein Data Bank (wwPDB) and the Electron Microscopy Data Bank (EMDB) with the following accession codes PDB: 8R4I and EMDB-18887.

## SUPPORTING MATERIAL

Supporting material can be found online at https://doi.org/10.1016/j.bpj.2024.02.009.

## AUTHOR CONTRIBUTIONS

D.V. and S.A.O. designed the study, and carried out all the cryo-EM screening and data processing. G.S. carried out surface tension measurements and ab initio calculations, and analyzed the data. H.C. produced peptide samples. A.A.T. facilitated laboratory equipment and helped design the study. M.M. designed the study, analyzed data, supervised all experimental work, and guided the preparation of the manuscript.

## ACKNOWLEDGMENTS

M.M. acknowledges the Swedish Research Council (grant no. VR 2020-05403), the Swedish Society for Medical Research (S20-0156), and Harald and Greta Jeanssons Foundation grant (J2021-0114). A.A.T. acknowledges the Knut and Alice Wallenberg Foundation via the Wallenberg Centre for Molecular and Translational Medicine and the Swedish Research Council (2020-04299). We acknowledge the use of the Cryo-EM Uppsala facility for grid preparation and cryo-EM screening, funded by the Department of Cell and Molecular Biology, the Disciplinary Domains of Science and Technology and of Medicine and Pharmacy at Uppsala University. These data were collected at the Cryo-EM Swedish National Facility funded by the Knut and Alice Wallenberg, Family Erling Persson and Kempe Foundations, SciLifeLab, Stockholm University, and Umeå University. The computations were enabled by resources provided by the National Academic Infrastructure for Supercomputing in Sweden (NAISS) at Uppsala University and Linköping University partially funded by the Swedish Research Council through grant agreement no. 2022-06725 (NAISS) and 2018-06479 (PReSTO). Computing time was provided to M.M. with the following grants: NAISS 2023/22-256, NAISS 2023/5-165, NAISS 2023/6-112, NAISS 2023/22-1272, Berzelius-2023-271, and Berzelius-2023-77.

## DECLARATION OF INTERESTS

The authors declare no competing interests.

## REFERENCES

1. Fitzpatrick, A. W., and H. R. Saibil. 2019. Cryo-EM of amyloid fibrils and cellular aggregates. *Curr. Opin. Struct. Biol.* 58:34–42.
2. Nogales, E. 2016. The development of cryo-EM into a mainstream structural biology technique. *Nat. Methods.* 13:24–27.
3. Bai, X.-C., G. McMullan, and S. H. W. Scheres. 2015. How cryo-EM is revolutionizing structural biology. *Trends Biochem. Sci.* 40:49–57.
4. Yip, K. M., N. Fischer, …, H. Stark. 2020. Atomic-resolution protein structure determination by cryo-EM. *Nature.* 587:157–161.
5. Lövestam, S., F. A. Koh, …, S. H. W. Scheres. 2022. Assembly of recombinant tau into filaments identical to those of Alzheimer's disease and chronic traumatic encephalopathy. *Elife.* 11, e76494.
6. Dobro, M. J., L. A. Melanson, …, A. W. McDowall. 2010. Plunge freezing for electron cryomicroscopy. *Methods Enzymol.* 481:63–82.
7. Iancu, C. V., W. F. Tivol, …, G. J. Jensen. 2006. Electron cryotomography sample preparation using the Vitrobot. *Nat. Protoc.* 1:2813–2819.
8. Cabra, V., and M. Samsó. 2015. Do's and don'ts of cryo-electron microscopy: a primer on sample preparation and high quality data collection for macromolecular 3D reconstruction. *J. Vis. Exp.* e52311.
9. Mills, D. J. 2021. Setting up and operating a cryo-EM laboratory. *Q. Rev. Biophys.* 54:e2.
10. Zielinski, M., C. Röder, and G. F. Schröder. 2021. Challenges in sample preparation and structure determination of amyloids by cryo-EM. *J. Biol. Chem.* 297, 100938.
11. Michon, B., U. López-Sánchez, …, M. Zoonens. 2023. Role of surfactants in electron cryo-microscopy film preparation. *Biophys. J.* 122:1846–1857.
12. Li, B., D. Zhu, …, X. Zhang. 2021. Effect of charge on protein preferred orientation at the air–water interface in cryo-electron microscopy. *J. Struct. Biol.* 213, 107783.
13. Egri, S. B., X. Wang, …, K. Shen. 2023. Detergent modulates the conformational equilibrium of SARS-CoV-2 Spike during cryo-EM structural determination. *Nat. Commun.* 14:2527.
14. Seddon, A. M., P. Curnow, and P. J. Booth. 2004. Membrane proteins, lipids and detergents: not just a soap opera. *Biochim. Biophys. Acta.* 1666:105–117.
15. Chen, S., J. Li, …, R. Henderson. 2022. Interaction of human erythrocyte catalase with air–water interface in cryoEM. *Microscopy.* 71:i51–i59.
16. Ratkeviciute, G., B. F. Cooper, and T. J. Knowles. 2021. Methods for the solubilisation of membrane proteins: the micelle-aneous world of membrane protein solubilisation. *Biochem. Soc. Trans.* 49:1763–1777.
17. Li, S. 2022. Detergents and alternatives in cryo-EM studies of membrane proteins: Detergents and alternatives in cryo-EM studies of membrane proteins. *Acta Biochim. Biophys. Sin.* 54:1049–1056.
18. Cao, E., M. Liao, …, D. Julius. 2013. TRPV1 structures in distinct conformations reveal activation mechanisms. *Nature.* 504:113–118.

19. Le Bon, C., B. Michon, …, M. Zoonens. 2021. Amphipathic environments for determining the structure of membrane proteins by single-particle electron cryo-microscopy. *Q. Rev. Biophys.* 54:e6.

20. Koehl, A., H. Hu, …, B. K. Kobilka. 2019. Structural insights into the activation of metabotropic glutamate receptors. *Nature.* 566:79–84.

21. Baker, M. R., G. Fan, and I. I. Serysheva. 2015. Single-particle cryo-EM of the ryanodine receptor channel in an aqueous environment. *Eur. J. Transl. Myol.* 25:35–48.

22. Lomont, J. P., K. L. Rich, …, M. T. Zanni. 2018. Spectroscopic signature for stable $\beta$-amyloid fibrils versus $\beta$-sheet-rich oligomers. *J. Phys. Chem. B.* 122:144–153.

23. Maj, M., J. P. Lomont, …, M. T. Zanni. 2018. Site-specific detection of protein secondary structure using 2D IR dihedral indexing: a proposed assembly mechanism of oligomeric hIAPP. *Chem. Sci.* 9:463–474.

24. Luca, S., W.-M. Yau, …, R. Tycko. 2007. Peptide conformation and supramolecular organization in amylin fibrils: constraints from solid-state NMR. *Biochemistry.* 46:13505–13522.

25. Cao, Q., D. R. Boyer, …, D. S. Eisenberg. 2021. Cryo-EM structures of hIAPP fibrils seeded by patient-extracted fibrils reveal new polymorphs and conserved fibril cores. *Nat. Struct. Mol. Biol.* 28:724–730.

26. Wöhr, T., F. Wahl, …, M. Mutter. 1996. Pseudo-prolines as a solubilizing, structure-disrupting protection technique in peptide synthesis. *J. Am. Chem. Soc.* 118:9218–9227.

27. Scheres, S. H. W. 2012. RELION: implementation of a Bayesian approach to cryo-EM structure determination. *J. Struct. Biol.* 180:519–530.

28. Becke, A. D. 1993. Density-functional thermochemistry. III. The role of exact exchange. *J. Chem. Phys.* 98:5648–5652.

29. Clark, T., J. Chandrasekhar, …, P. V. R. Schleyer. 1983. Efficient diffuse function-augmented basis sets for anion calculations. III. The 3-21+G basis set for first-row elements, Li–F. *J. Comput. Chem.* 4:294–301.

30. Francl, M. M., W. J. Pietro, …, J. A. Pople. 1982. Self-consistent molecular orbital methods. XXIII. A polarization-type basis set for second-row elements. *J. Chem. Phys.* 77:3654–3665.

31. Krishnan, R., J. S. Binkley, …, J. A. Pople. 1980. Self-consistent molecular orbital methods. XX. A basis set for correlated wave functions. *J. Chem. Phys.* 72:650–654.

32. McLean, A. D., and G. S. Chandler. 1980. Contracted Gaussian basis sets for molecular calculations. I. Second row atoms, Z=11–18. *J. Chem. Phys.* 72:5639–5648.

33. Spitznagel, G. W., T. Clark, …, W. J. Hehre. 1987. An evaluation of the performance of diffuse function-augmented basis sets for second row elements, Na-Cl. *J. Comput. Chem.* 8:1109–1116.

34. Frisch, M. e., G. Trucks, …et al., 2016. Gaussian 16, Revision C. 01.

35. Dennington, R., T. A. Keith, and J. M. Millam. 2019. GaussView Version 6. Semichem Inc. Shawnee Mission KS.

36. Buchanan, L. E., E. B. Dunkelberger, …, M. T. Zanni. 2013. Mechanism of IAPP amyloid fibril formation involves an intermediate with a transient $\beta$-sheet. *Proc. Natl. Acad. Sci. USA.* 110:19285–19290.

37. Ridgway, Z., X. Zhang, …, D. P. Raleigh. 2018. Analysis of the role of the conserved disulfide in amyloid formation by human islet amyloid polypeptide in homogeneous and heterogeneous environments. *Biochemistry.* 57:3065–3074.

38. Röder, C., T. Kupreichyk, …, G. F. Schröder. 2020. Cryo-EM structure of islet amyloid polypeptide fibrils reveals similarities with amyloid-$\beta$ fibrils. *Nat. Struct. Mol. Biol.* 27:660–667.

39. Zheng, S. Q., E. Palovcak, …, D. A. Agard. 2017. MotionCor2: anisotropic correction of beam-induced motion for improved cryo-electron microscopy. *Nat. Methods.* 14:331–332.

40. Rohou, A., and N. Grigorieff. 2015. CTFFIND4: Fast and accurate defocus estimation from electron micrographs. *J. Struct. Biol.* 192:216–221.

41. Bepler, T., A. Morin, …, B. Berger. 2019. Positive-unlabeled convolutional neural networks for particle picking in cryo-electron micrographs. *Nat. Methods.* 16:1153–1160.

42. Glaeser, R. M., B.-G. Han, …, J. H. D. Cate. 2016. Factors that influence the formation and stability of thin, cryo-EM specimens. *Biophys. J.* 110:749–755.

43. Glaeser, R. M. 2021. Preparing better samples for cryo–electron microscopy: Biochemical challenges do not end with isolation and purification. *Annu. Rev. Biochem.* 90:451–474.

44. Vasconcelos, M. T., M. A. Azenha, and O. M. Lage. 1996. Electrochemical Evidence of Surfactant Activity of the Hepes pH Buffer Which May Have Implications on Trace Metal Availability to Culturesin Vitro. *Anal. Biochem.* 241:248–253.

45. Babel, L., S. Bonnet-Gómez, and K. Fromm. 2020. Appropriate Buffers for Studying the Bioinorganic Chemistry of Silver(I). *Chemistry.* 2:193–202.

46. Edwardes Moore, E., S. J. Cobb, …, E. Reisner. 2022. Understanding the local chemical environment of bioelectrocatalysis. *Proc. Natl. Acad. Sci. USA.* 119, e2114097119.

47. Zhang, X., J. Liu, …, K. Huang. 2016. How the imidazole ring modulates amyloid formation of islet amyloid polypeptide: A chemical modification study. *Biochim. Biophys. Acta.* 1860:719–726.

48. Sledź, P., R. Kamiński, …, K. Woźniak. 2010. An experimental charge density of HEPES. *Acta Crystallogr. B.* 66:482–492.

49. Crennell, S. J., G. O. Hreggvidsson, and E. Nordberg Karlsson. 2002. The Structure of Rhodothermus marinus Cel12A, A Highly Thermostable Family 12 Endoglucanase, at 1.8Å Resolution. *J. Mol. Biol.* 320:883–897.

50. Maurice, F., I. Broutin, …, F. Dardel. 2008. Enzyme structural plasticity and the emergence of broad-spectrum antibiotic resistance. *EMBO Rep.* 9:344–349.

51. Zbacnik, T. J., R. E. Holcomb, …, M. C. Manning. 2017. Role of Buffers in Protein Formulations. *J. Pharmaceut. Sci.* 106:713–733.

52. Mao, Y., L. Yu, …, P. d. B. Harrington. 2016. New insights into side effect of solvents on the aggregation of human islet amyloid polypeptide 11–20. *Talanta.* 148:380–386.

53. Garnham, C. P., R. L. Campbell, …, P. L. Davies. 2011. Novel dimeric β-helical model of an ice nucleation protein with bridged active sites. *BMC Struct. Biol.* 11

54. Hudait, A., N. Odendahl, …, V. Molinero. 2018. Ice-Nucleating and Antifreeze Proteins Recognize Ice through a Diversity of Anchored Clathrate and Ice-like Motifs. *J. Am. Chem. Soc.* 140:4905–4912.

55. Eickhoff, L., K. Dreischmeier, …, T. Koop. 2019. Contrasting Behavior of Antifreeze Proteins: Ice Growth Inhibitors and Ice Nucleation Promoters. *J. Phys. Chem. Lett.* 10:966–972.

56. Qiu, Y., A. Hudait, and V. Molinero. 2019. How Size and Aggregation of Ice-Binding Proteins Control Their Ice Nucleation Efficiency. *J. Am. Chem. Soc.* 141:7439–7452.

57. Cascajo-Castresana, M., R. O. David, …, C. Marcolli. 2020. Protein aggregates nucleate ice: the example of apoferritin. *Atmos. Chem. Phys.* 20:3291–3315.

58. Engel, M. F. M., H. Yigittop, …, J. Antoinette Killian. 2006. Islet Amyloid Polypeptide Inserts into Phospholipid Monolayers as Monomer. *J. Mol. Biol.* 356:783–789.

59. Kampjut, D., J. Steiner, and L. A. Sazanov. 2021. Cryo-EM grid optimization for membrane proteins. *iScience.* 24, 102139.

60. Bloch, M., M. Santiveri, and N. M. I. Taylor. 2020. Membrane protein cryo-EM: cryo-Grid optimization and data collection with protein in detergent. *Methods Mol. Biol.* 2127:227–244.

61. Giusti, F., J.-L. Popot, and C. Tribet. 2012. Well-Defined Critical Association Concentration and Rapid Adsorption at the Air/Water Interface of a Short Amphiphilic Polymer, Amphipol A8-35: A Study by Förster Resonance Energy Transfer and Dynamic Surface Tension Measurements. *Langmuir.* 28:10372–10380.

62. Zhang, M., R. Hu, …, J. Zheng. 2014. Structural and energetic insight into the cross-seeding amyloid assemblies of human IAPP and rat IAPP. *J. Phys. Chem. B.* 118:7026–7036.
63. Cao, P., F. Meng, …, D. P. Raleigh. 2010. The ability of rodent islet amyloid polypeptide to inhibit amyloid formation by human islet amyloid polypeptide has important implications for the mechanism of amyloid formation and the design of inhibitors. *Biochemistry.* 49:872–881.
64. Wang, H., Z. Ridgway, …, D. P. Raleigh. 2015. Analysis of the ability of pramlintide to inhibit amyloid formation by human islet amyloid polypeptide reveals a balance between optimal recognition and reduced amyloidogenicity. *Biochemistry.* 54:6704–6711.
65. Liu, Y., D. T. Huynh, and T. O. Yeates. 2019. A 3.8 Å resolution cryo-EM structure of a small protein bound to an imaging scaffold. *Nat. Commun.* 10:1864.
66. Gallardo, R., M. G. Iadanza, …, N. A. Ranson. 2020. Fibril structures of diabetes-related amylin variants reveal a basis for surface-templated assembly. *Nat. Struct. Mol. Biol.* 27:1048–1056.
67. O'Nuallain, B., A. K. Thakur, …, R. Wetzel. 2006. Kinetics and thermodynamics of amyloid assembly using a high-performance liquid chromatography–based sedimentation assay. *Methods Enzymol.* 413:34–74.

Biophysical Journal, Volume *123*

# Supplemental information

## Improving cryo-EM grids for amyloid fibrils using interface-active solutions and spectator proteins

**Dylan Valli, Saik Ann Ooi, Giorgio Scattolini, Himanshu Chaudhary, Alesia A. Tietze, and Michał Maj**

## SUPPLEMENTARY MATERIAL

### Cryo-EM data processing

All movies were motion-corrected and dose-weighted using RELION(1) own implementation of UCSF MotionCor2(2). The contrast transfer function (CTF) was estimated with CTFFIND4.1(3). The 3dem implementation of Topaz(4) for fibrils was used to train an auto-picking model based on 100 manually picked micrographs and all fibrils' start-end coordinates were automatically picked over the whole dataset. The particles were extracted with a box size of 960 pixels down-sampled to 240 px and an inter-box distance of 3 asymmetrical units of 4.75 Å. Several rounds of 2D classifications were carried out to separate the polymorphs. The classes with the dominant polymorph were selected and the particles were re-extracted with a 600 px box down-sampled to 300 px for further 2D classifications. The best particles were extracted at 320 box size and further separated in a 3D classification job without symmetry using a cylinder generated with relion_helix_toolbox as an initial model. The main resulting class showed a typical C2 symmetry and the corresponding particles were used to perform another round of 3D classification job with imposed C2 symmetry. The final particles were run through a 3D-refine job using the previous 3D class as an initial model and a narrow search for helical symmetry. Both half-maps converged to similar values for twist and rise of 178 degrees and 2.45 Å respectively. Finally, the particles were polished and a post-process job was run to obtain the final map with a resolution of 4.01 Å. The resolution was calculated using gold-standard FSC at 0.143 threshold.

### Model building

The published PDB model (PDB:7M64) was used as an initial model and fitted into the cryo-EM map in COOT(5). The model was refined in COOT and real space refinement in PHENIX(6) iteratively. The model and map were visualized with UCSF ChimeraX(7). The cryo-EM data collection, refinement, and validation statistics are summarized in Table S2.

Table S1: Surface tension values for milli-Q water, 100 mM HEPES, 20 mM Tris-HCl and 2 mM SDS

| | $\gamma$ / $mNm^{-1}$ |
|---|---|
| milli-Q water | 72.3±0.3 |
| HEPES 100 mM | 71.4±0.3 |
| Tris-HCl 20 mM | 71.7±0.5 |
| SDS 2 mM | 62.6±1.1 |

Table S2: Data collection and refinement parameters for hIAPP-WT double S polymorph.

| | WT P1 (EMDB-18887) (PDB 8R4I) |
|---|---|
| **Data collection and processing** | |
| Magnification | 105 000 |
| Voltage (kV) | 300 |
| Electron exposure ($e^-$/Å$^2$) | 39.926 |
| Defocus range (µm) | 0.5 to 1.7 |
| Pixel size (Å) | 0.828 |
| Symmetry imposed | C2 |
| Twist (°) | 178.25 |
| Rise (Å) | 2.46 |
| Initial particles images (no.) | 189867 |
| Final particles images (no.) | 10566 |
| Map resolution (Å) | 4.01 |
| **Refinement** | |
| Initial model used (PDB code) | 7M64 |
| Model resolution (Å) | 4.01 |
| FSC threshold | 0.143 |
| Map sharpening B factor (Å$^2$) | |
| Protein | 56.48 |
| Ligand | 41.41 |
| **Model composition** | |
| Non-hydrogen atoms | 1850 |
| Proteins residues | 250 |
| Ligands | 10 |
| B factors (Å$^2$) | |
| **R.m.s.d.** | |
| Bond lengths (Å) | 0.005 |
| Bond angles (°) | 0.704 |
| **Validation** | |
| MolProbity score | 1.91 |
| Clashscore | |
| 26.55 Rotamers outlier (%) | 0 |
| **Ramachandran plot** | |
| Favored (%) | 100 |
| Allowed (%) | 0 |
| Disallowed (%) | 0 |

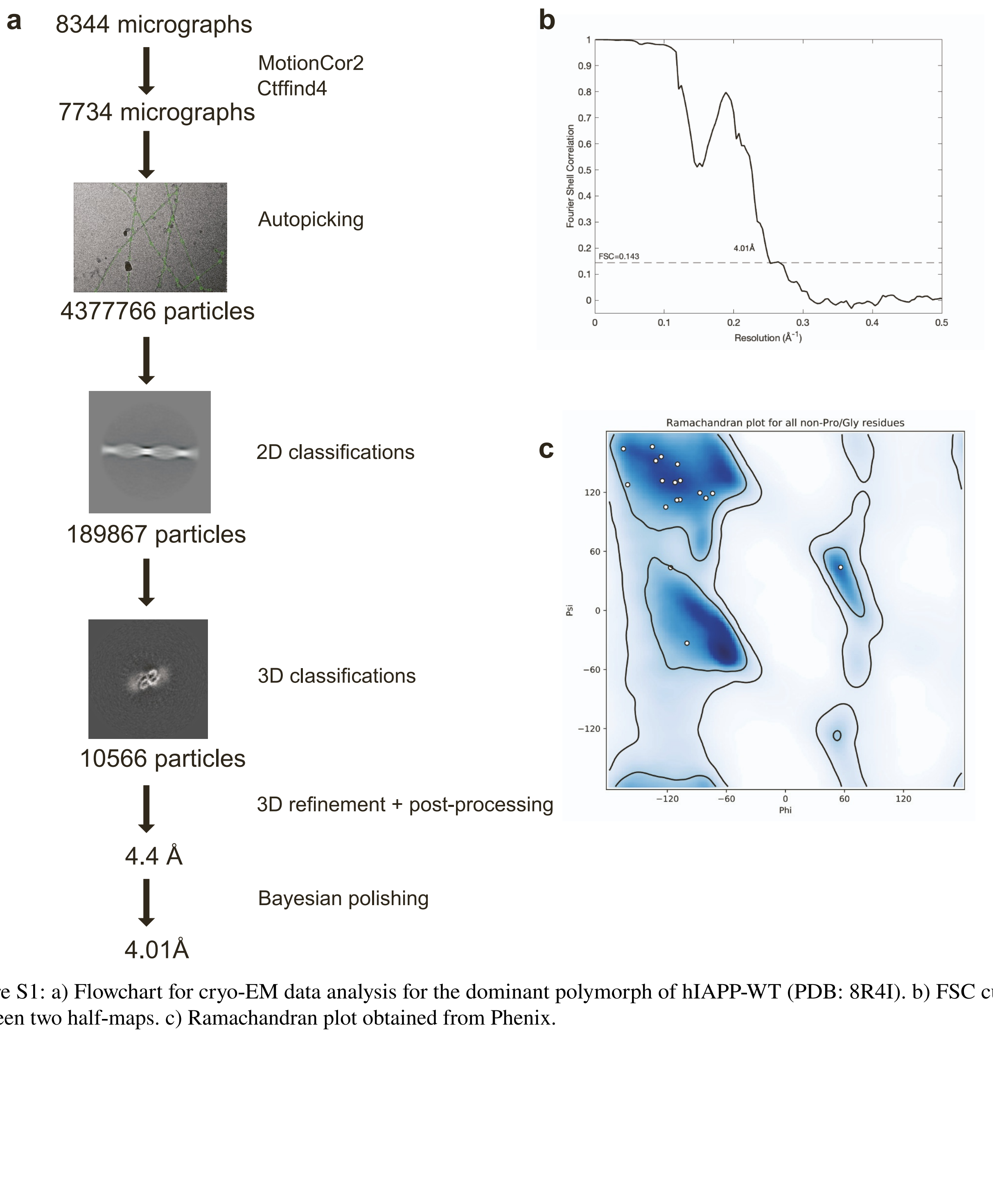


Figure S1: a) Flowchart for cryo-EM data analysis for the dominant polymorph of hIAPP-WT (PDB: 8R4I). b) FSC curves between two half-maps. c) Ramachandran plot obtained from Phenix.

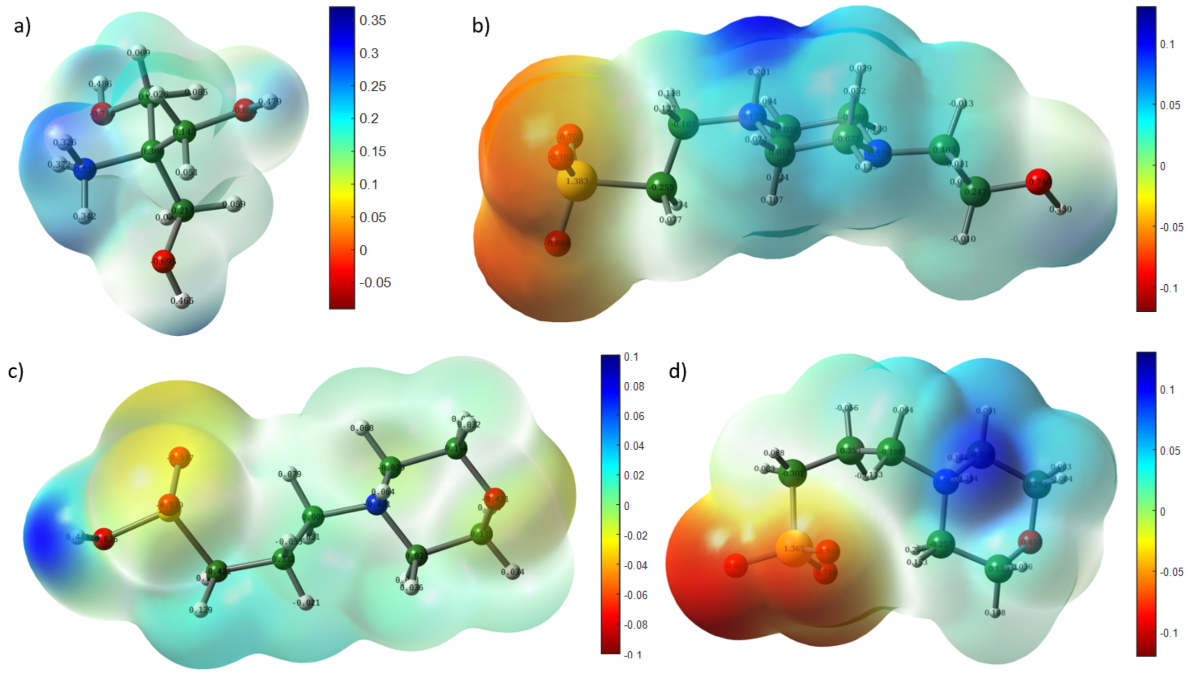


Figure S2: Electrostatic potential maps of a) Tris-HCl in its protonated form, b) HEPES in its zwitterionic form, c) MOPS in its neutral form and d) MOPS in its zwitterionic form.

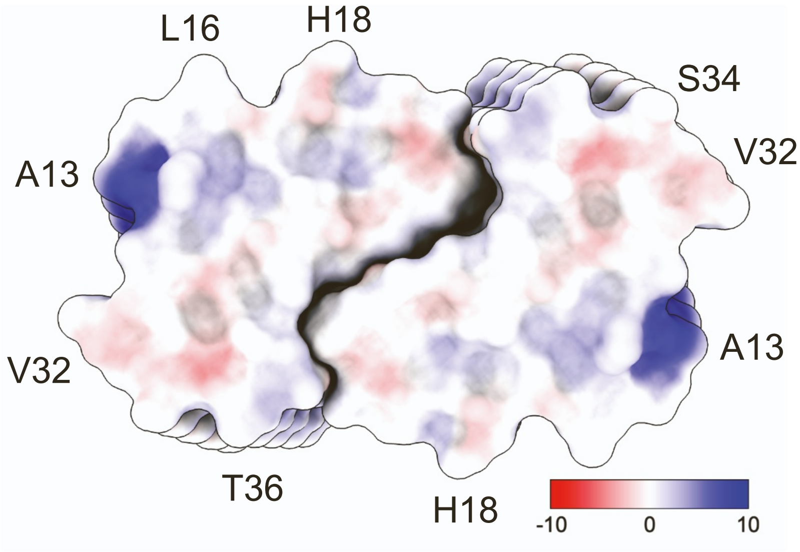


Figure S3: The electrostatic potential map of the solved WT-hIAPP structure. The map was generated with ChimeraX(8).

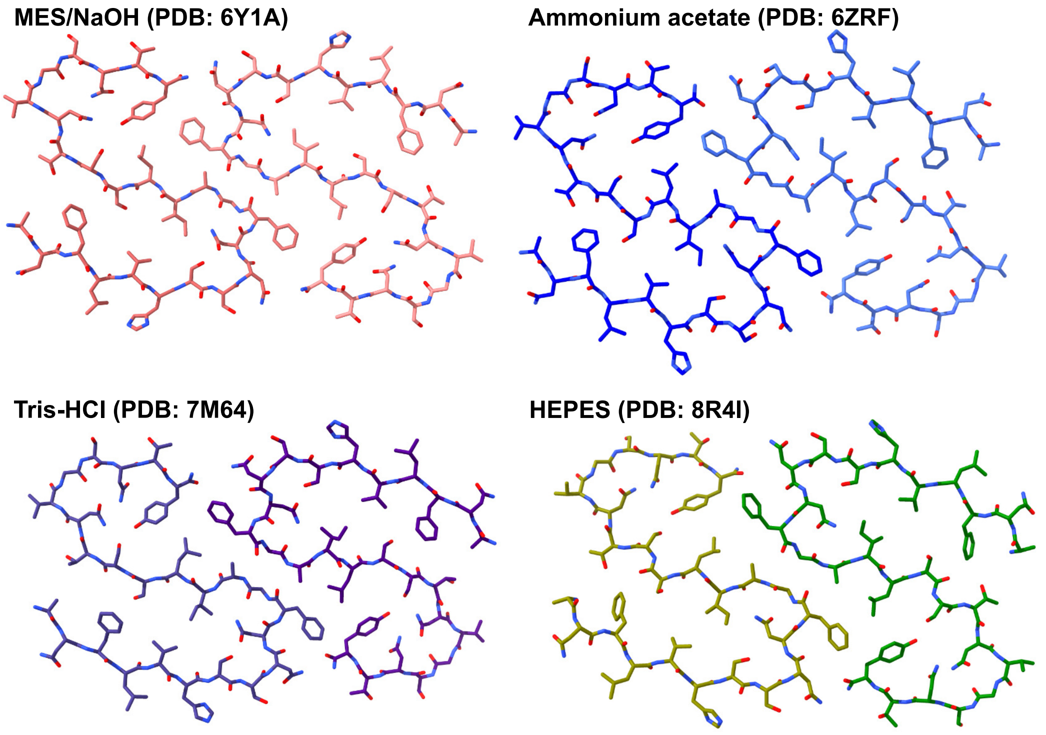


Figure S4: The dominant polymorph of hIAPP-WT aggregated in various buffer conditions. All the structures have almost identical backbone arrangements with exceptions in some rotameric amino acids.

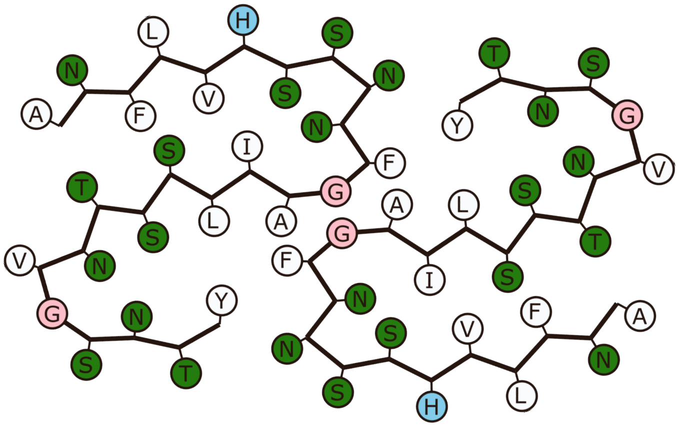


Figure S5: Schematic representation of hIAPP-WT, produced using atom2svg.py(9). The amino acids are color-coded according to their chemical properties. Positively charged residues are colored blue, polar residues are colored green, non-polar residues are colored white, and glycines are colored pink.

## REFERENCES


1. Scheres, S. H., 2012. RELION: implementation of a Bayesian approach to cryo-EM structure determination. *J. Struct. Biol.* 180:519–530.

2. Zheng, S. Q., E. Palovcak, J.-P. Armache, K. A. Verba, Y. Cheng, and D. A. Agard, 2017. MotionCor2: anisotropic correction of beam-induced motion for improved cryo-electron microscopy. *Nat. Methods* 14:331–332.

3. Rohou, A., and N. Grigorieff, 2015. CTFFIND4: Fast and accurate defocus estimation from electron micrographs. *J. Struct. Biol.* 192:216–221.

4. Bepler, T., A. Morin, M. Rapp, J. Brasch, L. Shapiro, A. J. Noble, and B. Berger, 2019. Positive-unlabeled convolutional neural networks for particle picking in cryo-electron micrographs. *Nat. Methods* 16:1153–1160.

5. Emsley, P., and K. Cowtan, 2004. Coot: model-building tools for molecular graphics. *Acta Cryst. D* 60:2126–2132.

6. Liebschner, D., P. V. Afonine, M. L. Baker, G. Bunkóczi, V. B. Chen, T. I. Croll, B. Hintze, L.-W. Hung, S. Jain, A. J. McCoy, et al., 2019. Macromolecular structure determination using X-rays, neutrons and electrons: recent developments in Phenix. *Acta Cryst. D* 75:861–877.

7. Pettersen, E. F., T. D. Goddard, C. C. Huang, E. C. Meng, G. S. Couch, T. I. Croll, J. H. Morris, and T. E. Ferrin, 2021. UCSF ChimeraX: Structure visualization for researchers, educators, and developers. *Protein Sci.* 30:70–82.

8. Meng, E. C., T. D. Goddard, E. F. Pettersen, G. S. Couch, Z. J. Pearson, J. H. Morris, and T. E. Ferrin, 2023. UCSF ChimeraX: Tools for structure building and analysis. *Protein Sci.* 32:e4792.

9. Nakane, T., version 210224. atom2svg. https://gist.github.com/biochem-fan/026ec2f191fee9285424d12fc2b84ce7.
